# The Acceptance of Postpartum Intrauterine Contraceptive Devices Among Women Who Receive Focused Family Planning Counseling in the Antenatal Period Compared to Those Who Receive Routine Counseling: A Randomized Controlled Trial

**DOI:** 10.7759/cureus.40344

**Published:** 2023-06-12

**Authors:** Ashwini Najan, Prachi Dixit, Anuja Bhalerao

**Affiliations:** 1 Department of Obstetrics and Gynaecology, NKP Salve Institute of Medical Sciences and Research Centre, Nagpur, IND

**Keywords:** tubal ligation, routine antenatal counseling, family planning, antenatal women, post-partum intrauterine contraceptive device

## Abstract

Background and objective

Unplanned pregnancies are very common in the postpartum period, and they often lead to negative outcomes such as abortion, low-birth-weight neonates, early delivery, postpartum bleeding, and fetal mortality. In the first 12 months after delivery, closely spaced and unintentional pregnancies can be prevented with postpartum contraception. The postpartum intrauterine contraceptive device (PPIUCD) is a method of family planning that may be used during the first few weeks after giving birth, and it is highly successful, reliable, affordable, non-hormonal, immediately reversible, long-acting, and does not interfere with lactation. Urban and educated women are largely aware of IUCD and its benefits, but the proportion of these women who use them is still small. In light of this, this study aimed to assess if providing focused antenatal counseling led to a greater postpartum IUCD acceptance rate when compared to routine counseling.

Method

We conducted a randomized controlled trial in the Department of Obstetrics and Gynaecology of a tertiary care center from January 2021 to December 2022. Based on the inclusion and exclusion criteria, 220 women were enrolled and were classified into two groups. Group A comprised 110 women (the focused counseling group) who received focused postpartum family planning (PPFP) counseling, and Group B consisted of the control group involving 110 women who received routine counseling.

Results

In both groups, the women who inserted IUCD were mostly gravida 2. Additionally, willingness to use IUCD was shown by 68% of women in the focused counseling group and 58% of women in the routine counseling group, and PPIUCD was accepted by 22% of women in the focused counseling group and 9% of women in the routine counseling group. Post-placental insertion was carried out in 18 (75%) cases in the focused counseling group and seven (70%) cases in the routine counseling group. Extended postpartum insertion (insertion within one year of delivery) was carried out in six (25%) cases in the focused counseling group and three (30%) cases in the routine counseling group. The most common reasons for the refusal were a preference for tubal ligation (TL) and a fear of side effects.

When the patients were enquired about their contraceptive use over the past year on telephone conversations after one year of delivery, it was observed that 21.81% inserted PPIUCD, 30% used injectable depot-medroxyprogesterone acetate (DMPA), 13.63% had undergone TL, 11.81% used barrier contraception, while 22.72% did not use any contraception in the focussed counseling group. In the routine counseling group, 16.36% of women inserted PPIUCD, 20% used injectable DMPA, 12.72% underwent TL, 18.18% used barrier contraception, and 32.72% did not use any contraception.

Conclusion

Although PPIUCD is a long-acting reversible contraceptive that is safe and reliable, only a few women choose it as a method of birth control. This may be due to ignorance, misconceptions, and worries/fears about potential difficulties/adverse effects associated with IUCD insertion. The IUCD stigma mostly results from misconceptions about the fear of complications. Hence, we recommend that proper IUCD counseling be provided during antenatal care visits to dispel misunderstandings and concerns regarding potential complications associated with PPIUCD insertion.

## Introduction

Unexpected and closely spaced pregnancies in the first year following delivery can be prevented using postpartum contraception. Unplanned pregnancies are very common in the postpartum period, and they often result in negative outcomes such as abortion, low-birth-weight neonates, preterm labor, postpartum bleeding, and fetal mortality [1]. Preterm birth, low birth weight, and small-for-gestational-age infants are all risk factors associated with pregnancies within the first year of motherhood [2]. In the first six months after giving birth, 37% of sexually active women are at risk of becoming pregnant, and that risk rises to 64% in the subsequent 6-11 months [3].

Before women resume their sexual activity or return to fertility after delivery, contraceptive techniques must be initiated to prevent unwanted pregnancies in the immediate postpartum period [4]. Hence, postpartum family planning (PPFP) is necessary, especially for women who have undergone cesarean surgery. The recognition of the need for providing women with effective contraceptive options immediately after childbirth has grown in recent years. By lengthening the birth gap, contraceptives could improve prenatal outcomes and child survival [5]. However, after giving birth, some women might choose not to use contraception right away, which could result in high-risk pregnancies and short birth intervals [3-6]. One reason for high rates of failure of postpartum family planning is the unavailability of counseling programs, which have to be typically provided during antenatal care, and the lack of willingness among couples [6].

The postpartum intrauterine contraceptive device (PPIUCD) is a method of family planning that may be used during the first few weeks after giving birth, and it is very successful, reliable, affordable, non-hormonal, immediately reversible, long-acting, and does not interfere with lactation. However, an intrauterine contraceptive device (IUCD) cannot be advised for women with a past history of menorrhagia or in cases of anemia, which is more prevalent [1]. Frequent medical appointments are not necessary with immediate IUCD insertion [7], and compared to interval insertion and delayed implantation after childbirth, it is simple and safe as even a mid-level-trained birth attendant can insert it [8,9]. Therefore, women should be motivated to accept family planning alternatives throughout the post-delivery period. Additionally, the husband or family should be involved during the counseling process [10].

For PPIUCD service providers, the antepartum period is a great time to offer the procedure, particularly in settings where women encounter societal or geographic barriers to accessing contraceptive services [11]. Postpartum IUCD usage is also significantly impacted by the male partner's rejection, religious convictions, and refusal of vasectomy. The risk of expulsion has historically been higher with post-placental and immediate postpartum insertion than with interval insertion; however, better insertion methods have decreased this risk [12]. Family welfare programs that focus on family planning constitute a crucial part of national healthcare initiatives. Despite coordinated and integrated efforts, these programs have not been able to significantly lower the crude birth rate [13].

While many urban and educated women are aware of IUCD, the proportion of these women who use them is still small, which reveals that even these women have several misunderstandings or fears related to the same. Another opportunity to meet the unfulfilled demand for planning a family after giving birth is provided through counseling on family planning throughout the antepartum period. By separating pregnancies by more than two years, family planning can reduce maternal and pediatric morbidity by more than 30% and 10%, respectively [14,15].

Focused prenatal family planning education increases the likelihood of postpartum women accepting family planning, as postpartum women are crucial target populations since they might not be aware that even breastfeeding women run the risk of getting pregnant [1]. Keeping all these objectives in mind, the present study was undertaken to assess if providing focused antenatal counseling led to a greater postpartum IUCD acceptance rate when compared to routine counseling. There is a dearth of studies from India comparing the acceptance of PPIUCD with focused counseling vs. routine counseling. There have been studies from central India that focus on the factors that affect the acceptance of PPIUCD but no study has been done as a follow-up to analyze the effects of antenatal counseling in addressing certain common factors and fears that primarily cause the decreased acceptance of PPIUCD.

The primary objective of the study was to compare the acceptance of PPIUCD in women who receive focused family planning counseling in the antenatal period as compared to those who receive routine counseling. The secondary objectives were to estimate the use of PPIUCD and other contraceptive methods in postpartum women up to one year after childbirth in tertiary healthcare and to study the complications related to PPIUCD use within one year of insertion.

## Materials and methods

A randomized controlled trial was conducted in the Department of Obstetrics and Gynaecology of a tertiary care center from January 2021 to December 2022 after obtaining approval from the Institutional Ethics Committee (IEC) with IEC reference number 104/2021. The inclusion criteria were as follows: women who attended the antenatal outpatient department for obstetric care and planned to deliver in the hospital with one or more opportunities for counseling at least one week before the delivery. The exclusion criteria were as follows: women who opted for permanent family planning methods; women with absolute or relative contraindications for IUCD insertion, such as previous ectopic pregnancy, fibroid complicating pregnancy, severe anemia, prolapse with pregnancy, and known allergy to copper. The withdrawal criteria consisted of patients who developed any complication during delivery that made them unfit for PPIUCD, including rupture of membranes, chorioamnionitis, puerperal sepsis, genital trauma, and postpartum hemorrhage.

Based on the above-mentioned inclusion and exclusion criteria, 220 women from central India were included in the study, and classified into two groups by block randomization. Group A, which was considered the intervention group, included 110 antenatal women who were directed towards family planning OPD and received PPFP counseling using the standard cue cards as the counseling tool. The antenatal cards of the patient were stamped after PPFP counseling for easy identification on further visits and during labor. Additionally, the counseling was repeated during further visits and intrapartum periods. Group B including 110 antenatal women was considered the control group in which PPFP counseling was done based on a routine hospital approach, at bedside post-delivery or intrapartum, without any antenatal diversion towards family planning OPD. Sociodemographic details along with the obstetric, menstrual, and other relevant medical history were noted. Patients were followed up during antenatal visits as per standard hospital protocols. The decision of women regarding the choice of contraceptive was recorded. The service for post-placental, immediate, or interval IUCD was provided to the patient by the hospital if they opted for IUCD insertion. Both the intervention group and control group were followed up for one year post-delivery via telephone to gather information about the acceptance of IUCD or another contraceptive method.

The data were entered into a Microsoft Excel sheet. IBM SPSS Statistics 21.0 (IBM Corp., Armonk, NY) for Windows was used to statistically analyze the data. For categorical data, frequencies and percentages were calculated, and for quantitative data, the mean was calculated; for inferential statistics, the Chi-square test was applied. The significance of the difference in the distribution of categorical variables across two study groups was tested using the Chi-square test or Fisher’s exact probability test. Whereas, to determine the significance of the difference in the means of continuous variables between the two research groups, an independent sample t-test was performed. A p-value below 0.05 was considered statistically significant.

## Results

In the current study, the average age of the patients was 27.64 ± 6.40 years in the focused counseling group and 27 ± 6.94 years in the routine counseling group. The highest number of patients were in the age group of 21-30 years (n=65, 60%) in the focused counseling group and 56 (50%) in the routine counseling group followed by 32 (28%) in the age group of 31-40 years in focused counseling group and 36 (34%) in the routine counseling group. As for the distribution of women according to age group, most of the women in both the focused counseling group and routine counseling group who accepted PPIUCD belonged to the age group of 21-30 years. Overall, 24 PPIUCDs were inserted in the focused counseling group and 10 in the routine counseling group. The majority of the patients (65%) hailed from rural areas and were almost equally distributed in both groups, with 72 in the focused counseling group and 71 in the routine counseling group.

Out of the total women who received counseling, more women from urban areas accepted PPIUCD in the focused counseling group (n=10/38 26.31%) and in the routine counseling group (8/39, 20.52%); whereas among women from rural areas, the acceptance was relatively low: 19.44% in the focused counseling group and 11.26% in the routine counseling group. Of the total 110 women from each group, the majority of individuals belonged to the lower socioeconomic class, with 58 (58%) in the focused counseling group and 74 (66%) in the routine counseling group, followed by those in the lower middle class, with 35 (32%) in the focused counseling group and 15 (14%) in the routine counseling group. However, even though the majority of women belonged to the lower class, the proportion of women who underwent counseling and accepted PPIUCD from the upper class was higher when compared to their counterparts in the lower classes.

Those who had completed only primary education constituted the highest number of patients (n=53, 48%) in the focused counseling group and in the routine counseling group (n=73, 66%), while 48 (44%) patients in the focused counseling group and 30 (28%) in the routine counseling group had completed secondary education. The acceptance of PPIUCD in both the focused counseling group and the routine counseling group was more prevalent among women who were graduates or had completed their secondary education. The majority of participants were homemakers in both groups: 102 (92%) in the focused counseling group and 106 (97%) in the routine counseling group. While there were only a few working women in both groups, a higher proportion of them accepted PPIUCD compared to their non-working counterparts in both groups: two out of eight (25%) in the focused counseling group and two out of four (50%) in the routine counseling group.

The distribution of cases according to the obstetric history of those who inserted PPIUCD is presented in Table 1.

**Table 1:** Distribution of cases according to the obstetric history of those who inserted PPIUCD PPIUCD: postpartum intrauterine contraceptive device

Obstetric history	Group A		Group B	
	Counseled	Total inserted	Counseled	Total inserted
Primigravida	32 (100%)	5 (15.62%)	30 (14%)	3 (10%)
Gravida 2	37 (100%)	11 (29.72%)	42 (38.18%)	4 (18.18%)
Gravida 3	22 (100%)	4 (18.18%)	25 (11%)	2 (8%)
Gravida 4	15 (100%)	3 (20%)	10 (5%)	1 (10%)
Gravida 5	4 (100%)	1 (25%)	3 (1%)	0 (0%)
Total	110	24	110	10

The distribution of cases according to the willingness for IUCD insertion during counseling is shown in Table 2.

**Table 2:** Distribution of cases according to willingness for IUCD insertion during counseling IUCD: intrauterine contraceptive device; NS: not significant

Willingness for IUCD insertion during counseling	Group A, n (%)	Group B, n (%)	Total, n	P-value
Willing	74 (68%)	63 (58%)	137	0.126 (NS)
Declined	36 (32%)	47 (42%)	83	
Total	110 (100%)	110 (100%)	220	-

The distribution of cases according to the acceptance of PPIUCD is demonstrated in Table 3.

**Table 3:** Acceptance of PPIUCD PPIUCD: postpartum intrauterine contraceptive device; S: significant

Follow-up status	Group A, n (%)	Group B, n (%)	Total, n	P-value
IUCD inserted	24 (22%)	10 (9%)	34	0.009 (S)
Denied	86 (78%)	100 (91%)	178	
Total	110 (100%)	110 (50%)	220	-

**Figure 1:**
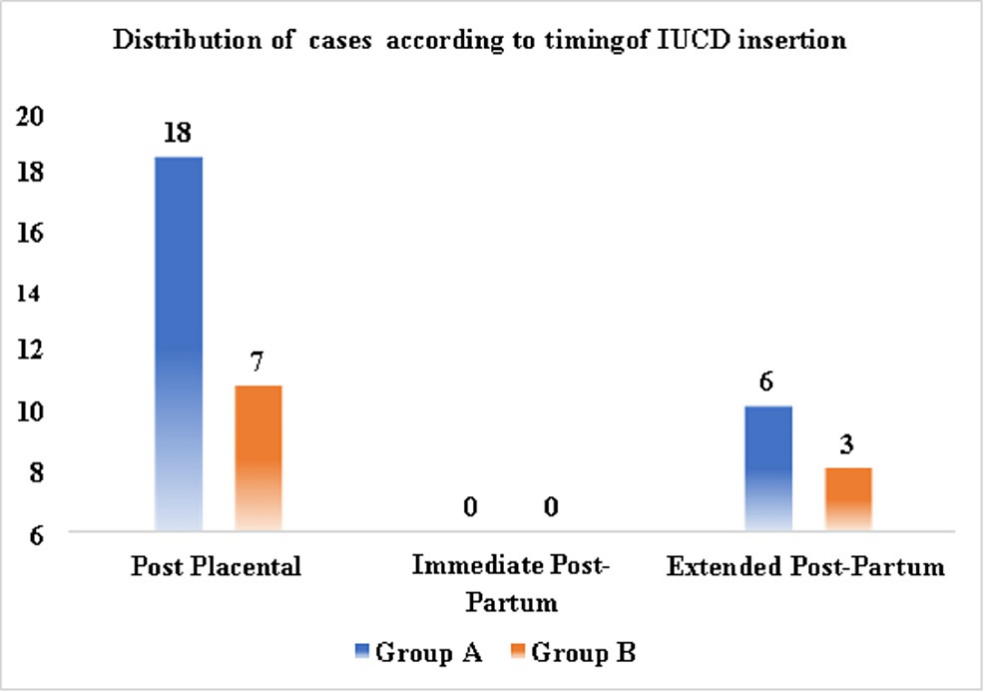
Distribution of cases according to the timing of IUCD insertion IUCD: intrauterine contraceptive device

Figure 1 shows the distribution of cases according to the timing of IUCD insertion.

A comparison between the willingness for PPIUCD insertion and actual acceptance in the cohort is demonstrated in Table 4.

**Table 4:** Comparison of the willingness for PPIUCD insertion with actual acceptance in the cohort PPIUCD: postpartum intrauterine contraceptive device

Group A, n (%)			Group B, n (%)		
Counseled	Willing	Accepted	Counseled	Willing	Accepted
110 (50%)	74 (34%)	24 (11%)	110 (50%)	63 (29%)	10 (9%)

Table 5 shows the distribution of cases according to the reason for the refusal of PPIUCD.

**Table 5:** Distribution of cases according to the reason for refusal of PPIUCD PPIUCD: postpartum intrauterine contraceptive device; TL: tubal ligation

Reason for refusal	Group A, n (%)	Group B, n (%)	Total, n (%)
Preference for TL	41 (47.67%)	40 (40%)	81 (43.55%)
Fear of side effects	35 (40.70%)	46 (46%)	81 (43.55%)
Opposition from partner	4 (4.65%)	6 (6%)	10 (5.37%)
Religious reasons	2 (2.32%)	4 (4%)	6 (3.22%)
Others (not willing to share reason)	4 (4.65%)	4 (4%)	8 (4.30%)
Total	86 (100%)	100 (100%)	186

The distribution of women according to the choice of contraceptive use in the postpartum period is demonstrated in Table 6.

**Table 6:** Choice of contraception among women in the study in the postpartum period DMPA: depot medroxyprogesterone acetate; POP: progestogen-only pill; PPIUCD: postpartum intrauterine contraceptive device; TL: tubal ligation

	Total women counseled	Women who inserted PPIUCD	Women who used inj. DMPA	Women who underwent TL	Women who used the barrier method	Women who used POP	Women who used implants	Women who did not use any contraception
Group A, n (%)	110 (100%)	24 (21.81%)	33 (30%)	15 (13.63%)	13 (11.81%)	0 (0%)	0 (0%)	25 (22.72%)
Group B, n (%)	110 (100%)	10 (9%)	22 (20%)	14 (12.72%)	20 (18.18%)	0 (0%)	0 (0%)	44 (40%)

The complications related to IUCD among women in the study are summarized in Table 7.

**Table 7:** Distribution of cases according to post-IUCD insertion complications IUCD: intrauterine contraceptive device

Post-IUCD complications	Group A, n (%)	Group B, n (%)	Total, n (%)
Menorrhagia	0 (0%)	1 (10%)	1 (2.94%)
Intermenstrual spotting	1 (4%)	1 (10%)	2 (5.88%)
Lower abdominal pain	1 (4%)	1 (10%)	2 (5.88%)
Absent	22 (92%)	7 (70%)	29 (85.29%)
Total	24 (100%)	10 (100%)	34 (100%)

## Discussion

The present study was conducted in the Department of Obstetrics and Gynaecology from January 1, 2021, to December 31, 2022, to assess the acceptance of PPIUCD in women who receive focused family planning counseling in the antenatal period as compared to those who receive routine counseling. A total of 220 antenatal women were enrolled, of which 110 underwent focused family planning counseling and 110 women underwent routine counseling. It was found that 24 patients from group A and 10 patients from group B eventually inserted PPIUCD. Both the intervention group and non-intervention group were followed up at one year via telephone to verify their acceptance of IUCD or another contraceptive method.

On analyzing the demographic profile of the patients, it was found that the mean age of the patients was 27.64 ± 6.40 years in the focused counseling group and 27 ± 6.94 years in the routine counseling group (range: 18-40 years). The highest number of patients were in the age group of 21-30 years in both groups: 65 (60%) in the focused counseling group and 56 (50%) in the routine counseling group. This finding is almost comparable to the mean age of 25.6 ± 3 years in the focused counseling group and 26.5 ± 3.7 years in the routine counseling group in a study conducted by Agarwal et al. with 264 participants in the focused counseling group and 154 in the routine counseling group [16]. Another similar study by Raghuwanshi et al. showed the average age of the population to be between 21 and 35 years [17].

Additionally, the majority of the patients (65%) hailed from rural areas and were almost equally distributed in both groups, with 72 in the focused counseling group and 71 in the routine counseling group. Out of the total women who received counseling, more women from urban areas accepted PPIUCD: 10/38 (26.31%) in the focused counseling group and 8/39 (20.52%) in the routine counseling group. In contrast, among women from rural areas, the acceptance was relatively low: 19.44% in the focused counseling group and 11.26% in the routine counseling group. Wayessa et al. performed a study in which it was seen that 45.5% of participants from the non-intervention group (n=484) and 65.2% from the intervention group (n=242) resided in urban areas [18].

According to the modified Kuppuswamy scale, of the total 110 women from each group, the majority of individuals belonged to the lower socioeconomic class, with 58 (58%) in the focused counseling group and 74 (66%) in the routine counseling group, followed by those in the lower middle class, with 35 (32%) in focused counseling group and 15 (14%) in the routine counseling group. However, even though the majority of women belonged to the lower class, the proportion of women who underwent counseling and accepted PPIUCD from the upper class was higher when compared to their counterparts in the lower classes. In a study by Agarwal et al., the majority of women belonged to the upper class (57.2%), which is in contrast with the present study where the majority of patients were from the lower class; however, the acceptance of IUCD was observed to be more in the upper class in the Agarwal study, which correlates with the findings of the present study [16].

As for the religion of the study population, out of 220 women, most women were Hindu: 69 (62%) in the focused counseling group and 64 (58%) in the routine counseling group, followed by Muslims: 37 (34%) in focused counseling group and 39 (36%) routine counseling group. When acceptance of PPIUCD was studied religion-wise, more proportion of women belonging to the Muslim religion accepted PPIUCD. These findings are in line with those of a study by Gadre et al., where 91% of the participants were Hindus, 8% were Muslims, and 1% belonged to other religions [19]. Similarly, in a study by Agarwal et al., the majority of the women were found to be Hindus, with 80.1% in the focused counseling group and 81.2% in the routine counseling group [16]. In their study also, more proportion of Muslim women accepted PPIUCD (n=8/29, 27.8%) as compared to Hindu women (n=28/125, 22%). The higher rate of acceptance of PPIUCD in Muslim women as compared to Hindu women might be due to religious reasons; of note, fewer Muslim women accept tubal ligation (TL) and resort to temporary solutions like IUCD instead.

Those who had completed only primary education constituted the highest number of patients (n=53, 48%) in the focused counseling group and in the routine counseling group (n=73, 66%), while 48 (44%) patients in the focused counseling group and 30 (28%) in the routine counseling group had completed secondary education. The acceptance of PPIUCD in both the focused counseling group and the routine counseling group was more prevalent among women who were graduates or had completed their secondary education. These results correlated well with the study by Agarwal et al. where most of the participants were illiterate or had only primary education: 65% in the focused counseling group and 75% in the routine counseling group [16]. Additionally, in a study conducted by Raghuwanshi et al., 71.5% of women had received only primary education [17]. The similar distribution of education among women in all three studies could be attributed to the fact that these studies were conducted in central and north India and involved participants with similar sociodemographic profiles. Wayessa et al. reported that 13.7% of the interventional group and 14.5% of the non-interventional group had no formal education, which was much higher than the findings of the present study, where only 1% in the interventional group and 3% in the non-interventional group lacked any formal education [18]. Also, the study by Tafere et al., which involved a total of 823 sample sizes out of which 187 participants received counseling and 636 did not, observed that 20.8% of the total participants were illiterate [20].

The majority of participants were homemakers (94.55%). While there were only a few working women in both groups, a higher proportion of them accepted PPIUCD compared to their non-working counterparts in both groups: two out of eight (25%) in the focused counseling group and two out of four (50%) in the routine counseling group. Wayessa et al. reported that most of the participants in their study were homemakers (56.86%); however, the rate of insertion was higher in working women (10.6%) than in homemakers (4.8%), which aligns with the present study [18]. Working women across the world are more independent decision-makers as compared to homemakers, which explains the higher rate of acceptance of PPIUCD among working women.

In the present study, the majority of participants were gravida 2 (n=79, 36%): 37 belonged to Group A and 42 belonged to Group B. However, in a study by Agarwal et al., the majority of the participants were primigravida: 57.6% in Group A and 79.9% in Group B, followed by gravida 2: 22.7% belonging to Group A and 29.2% belonging to Group B [16]. Also, a study conducted by Gadre et al. showed similar results with most of the participants being primigravida (31.84%), followed by 31.09%, 22.16%, 7.12%, 5.24%, and 2.62% of the women being gravida 2, gravida 3, gravida 4, gravida 5, and gravida 6, respectively [19]. The mean age in the present study and the above-mentioned studies were similar; however, the majority of the women were primigravida in the other studies, whereas the majority was gravid 2 in the present study, which could be due to the relatively early age of marriage among women in the region.

On analyzing the association between the acceptance of PPIUCD and obstetric history in the present study, it was found that in Group A the majority of women who accepted PPIUCD were gravida 2 whereas no such association of parity with acceptance was seen in Group B. In the study by Wayessa et al. also, the majority of the women who accepted PPIUCD were gravida 2 or more. Additionally, in the present study and Wayessa et al. study, many gravida 4 and 5 women also opted for PPIUCD rather than going for the permanent method of contraception [18].

Willingness for IUCD insertion during counseling was shown by 74 out of 110 (68%) women in Group A and 63 (58%) in Group B, whereas 36 (32%) in Group A and 47 (42%) in Group B did not show willingness for IUCD insertion. Similarly, in a study by Agarwal et al., 70.5% of the participants in Group A and 29.5% in Group B showed a willingness for PPIUCD insertion [16]. In the present study, 24 (22%) women in Group A and 10 (9%) women in Group B accepted PPIUCD whereas 86 (78%) in Group A and 100 (91%) in Group B refused at the time of insertion even if they had shown willingness for insertion previously. This correlated well with a study by Wayessa et al., which showed 12.4% and 4.8% of insertion of PPIUCD in the interventional and non-interventional groups, respectively [18]. Additionally, research conducted by Tafere et al. demonstrated that 12.4% of participants in Group A and 4.8% in Group B inserted PPIUCD [20].

In general, it was observed that the overall acceptance of PPIUCD either by those who underwent focused counseling or by those who received casual or no counseling was less than targeted. Still, in all of the above studies mentioned, the acceptance of PPIUCD after focused counseling is significantly higher than in the non-focused counseling group. In contrast to these studies, a few other studies had much higher acceptance in both groups, but again the acceptance in the focused counseling group was significantly higher than in the non-focused group. These include a study by Agarwal et al. in which it was observed that 116 (43.9%) of 264 participants in Group A and 36 (23.4%) of 154 participants in Group B inserted PPIUCD, respectively [16]. Raghuwanshi et al. in their study stated that the proportion of participants who inserted PPIUCD was 36.8% in Group A and 15.1% in Group B [17]. However, a relatively higher proportion of 78% in the intensive counseling group and 66% in the routine counseling group was observed in a study by Ndegwa et al., which aimed to determine the outcome of two levels of counseling on the utilization of post-placental PPIUCD [21].

Post-placental insertion was carried out in 18 (75%) cases in Group A and seven (70%) cases in Group B. Extended postpartum insertion (insertion within one year of delivery) was carried out in six (25%) cases in Group A and three (30%) cases in Group B. Thus, it was observed that it is in the immediate post-placental time when the patient is most receptive and hence optimal efforts should be taken to reinforce the need for immediate postpartum contraceptive protection when the patient presents for delivery.

On comparing the willingness to insert PPIUCD with actual acceptance, it was observed that the number of women who showed willingness was much higher than the number of women who actually accepted PPIUCD. When the reason for refusal was analyzed, it was seen that the highest number of cases [41 (47.67%) in Group A and 40 (40%) in Group B] showed a preference for TL, and an almost similar number of women reported fear of side effects, which led to the refusal. Also, 5.3% of women faced opposition from their partner or family, 3.2% of women quoted religious reasons, and 4.3% had no specific reason. Similarly, a study by Agarwal et al. reported that the reason for refusal of PPIUCD was mostly due to refusal on the part of the family (62.7%), while most of the participants had no reason or were not willing to share the reason for refusal and 11.9% of the participants did not have enough knowledge about PPIUCD [16]. In our study, no complications were seen in the majority of cases. However, menorrhagia was found in one case in Group B, intermenstrual spotting in one case each in Group A and Group B, and lower abdominal pain in one case each in Group A and Group B. Similarly, in a study by Ndegwa et al., very few participants (5%) experienced complications: expulsion was seen in 4.1% and abdominal pain in 1.8% of the participants [21]. Additionally, a study done by Rani et al. reported that 15% of participants experienced lower abdominal pain, 2.28% had menorrhagia, 5.48% had irregular vaginal bleeding, and 8.21% had cervicogenic discharge [22].

In the present study, while fear of side effects was one of the major factors quoted as a reason for refusal of PPIUCD, it was seen that the incidence of complications related to PPIUCD was very low. There were no major complications; only 14% of patients had mild lower abdominal pain or intermenstrual spotting and only 1% of patients had significant dysmenorrhea. Hence, in conclusion, focused family planning counseling performed in antenatal OPDs using standard cue cards and its reinforcement in each antenatal visit proved to be beneficial in terms of the acceptance of IUCD among women.

## Conclusions

Even though PPIUCD is a safe and long-acting reversible contraceptive, the acceptance of PPIUCD as a choice of contraception is low among women. This might be due to a lack of awareness, especially in the rural population, and perceived concerns and fears of complications related to IUCD insertion. The male partner’s and family's refusal as well as religious beliefs also play a significant role in the usage of postpartum IUCDs. However, when focused counseling was conducted using systematic charts and models, the acceptance of PPIUCD increased significantly as compared to routine counseling methods. Furthermore, when the women were followed up for one year, it was seen that most of the women who received counseling preferred injectable DMPA over PPIUCD. TL was another preferred method among those with an extended family. Even after many sessions of contraceptive counseling, 34.5% of women did not use any contraception or used barrier contraception, whereas 30% of women utilized injectable DMPA as the contraception of choice. The popularity of DMPA may be attributed to the fact that is injectable and does not need follow-up. However, If the patient is sufficiently educated about the importance of follow-up and if their fears/concerns about PPIUCD are properly addressed by providing assurance during follow-up visits, we may witness an improvement in their attitude toward PPIUCD.

Since the reason behind not accepting IUCD is mostly due to misconceptions about fear of complications, effective IUCD-related counseling should be done during antenatal care visits to correct these misconceptions and fears of complications about PPIUCD insertion. More awareness programs should be conducted for women and families regarding contraceptive methods. Women with higher levels of education and working women were more receptive toward immediate PPIUCD use. Therefore, adequate and appropriate attention should be given to enhancing the educational level of women and making them financially self-dependent for effective decision-making.
